# The pathogenesis and targeted therapies of intervertebral disc degeneration induced by cartilage endplate inflammation

**DOI:** 10.3389/fcell.2024.1492870

**Published:** 2024-12-02

**Authors:** Hantao Yang, Xuandu Chen, Jun Chen, Yansong Dong, Yafang Huang, Lei Qin, Jie Tan, Weihong Yi

**Affiliations:** ^1^ Department of Spine Surgery and Innovative Laboratory of Orthopedics, Shenzhen Nanshan People’s Hospital, Shenzhen, Guangdong, China; ^2^ Orthopedic Laboratory, Orthopedic Department and Hubei Sports Medicine Center, Wuhan Fourth Hospital, Wuhan, China

**Keywords:** intervertebral disc degeneration, cartilage endplate, inflammation, exosomes, calcification, ferroptosis, senescence

## Abstract

Intervertebral disc degeneration (IVDD) is the leading cause of low back pain, where degeneration and death of nucleus pulposus cells within the intervertebral disc (IVD) can be obviously revealed. This degeneration can result in an imbalance in the extracellular matrix due to the loss of proteoglycans and water content, which can further lead to catabolic and anabolic dysfunction of the IVD. Recently, the dysfunction of cartilage endplate (CEP) during aging has drawn large attention due to its essential functions in contributing nutrient exchange and maintaining IVD homeostasis. Furthermore, the inflammation and disturbed homeostasis of CEP not only accelerate the degradation of nucleus pulposus extracellular matrix, but also exacerbate IVDD by causing nucleus pulposus cell death through other pathological factors. Here in this review, we summarized the possible pathological factors and the underlying mechanisms of the CEP inflammation-induced IVDD, including exosomes degeneration, CEP calcification, ferroptosis, mechanical changes, and cell senescence. Besides, changes of miRNAs, pain-related neural reflex arc and pathways associated with CEP inflammation-induced IVDD are also reviewed. In addition, new strategies specifically designed for CEP inflammation-induced IVDD are also discussed in the last section. We hope this paper can not only offer some new insights for advancing novel strategies for treating IVDD, but also serve as a valuable reference for researchers in this field.

## 1 Introduction

Intervertebral disc (IVD) is a kind of fibrocartilaginous joint that is composed by a central nucleus pulposus (NP) in the center and a peripheral annulus fibrosus (AF) around the NP, while the cartilage endplate (CEP) is located between the intervertebral disc and the vertebral body (Dowdell et al., 2017). Functionally, the IVD plays important roles in bearing weight, absorbing shock, buffering cushion, and maintaining stability of the spine. However, intervertebral disc degeneration (IVDD) is recognized as the culprit of low back pain, which greatly reduces the quality of human life (Maher et al., 2017). The development of IVDD is a complex pathological process involving multiple factors, including extracellular matrix (ECM) depletion, fibration and dehydration of nucleus pulposus, extensive CEP injury, and subchondral sclerosis, which jointly decrease the height of intervertebral disc (Che et al., 2020; Lu et al., 2020; Tang et al., 2019). Besides, elevated inflammatory cytokines such as IL-1 and TNF-α in the IVD are revealed to be closely associated with disc degeneration, where IL-1 is reported to directly mediate various pro-inflammatory mediators and matrix metalloproteinases (MMPs), resulting in a disturbed ECM metabolism of the intervertebral disc (Gao et al., 2024). While the activation of TNF-α can lead to the expansion of inflammation cascades, and apoptosis of nucleus pulposus cells (NPCs) can be induced through its corresponding receptors of TNFR in such inflammatory circumstance, which will further result in spine dysfunction if left untreated (Alkhatib et al., 2014; Chen et al., 2017; Kang et al., 2015; Sutovsky et al., 2017).

**FIGURE 1:**
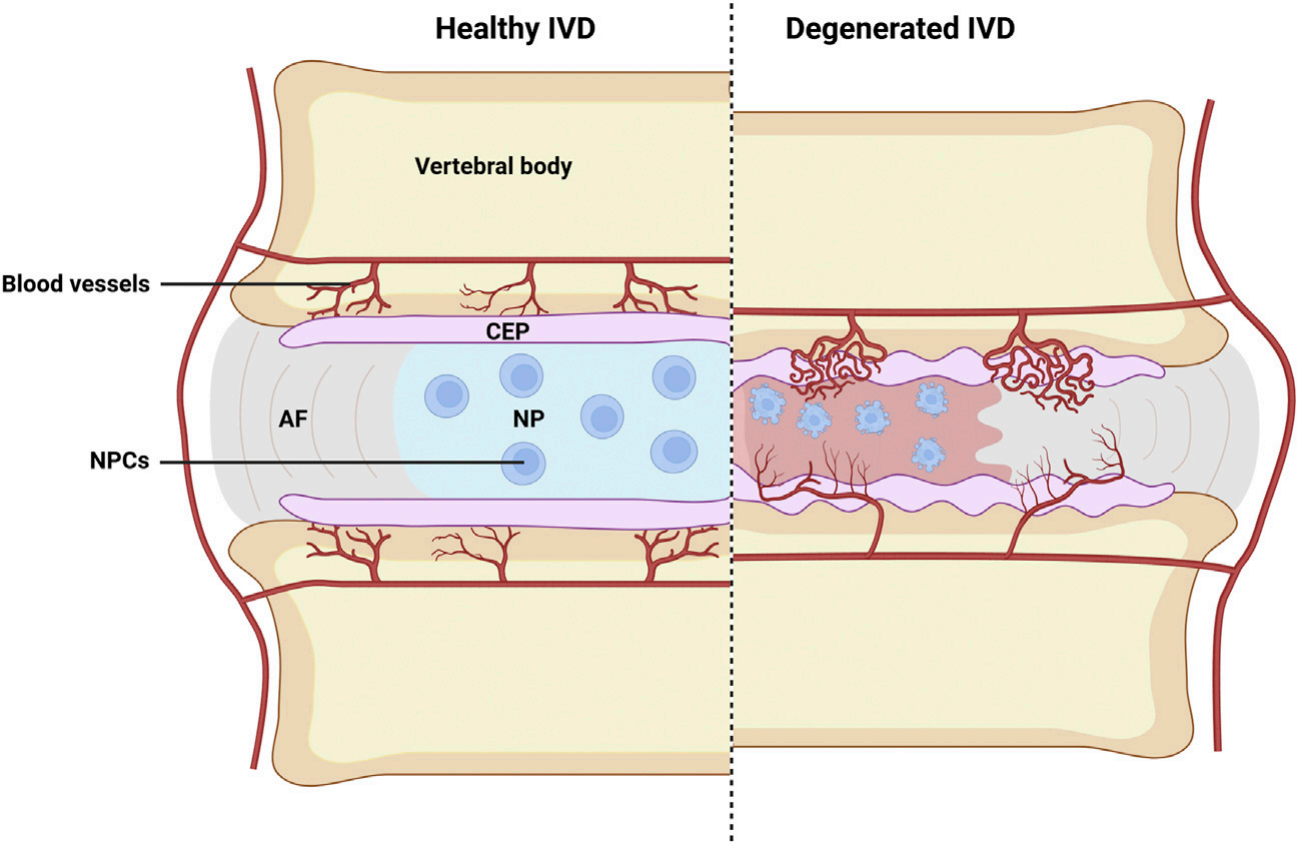
Schematic illustration of healthy and degenerated IVD. Where CEP destruction, abnormal infiltration of blood vessels, inflammation of nucleus pulposus, and evident NP cells apoptosis can all be observed in degenerated IVD. AF, fibrous rings; NP, nucleus pulposus; CEP, cartilage endplate; NPCs, nucleus pulposus cells. Image created with BioRender (www.biorender.com).

Cartilage endplate (CEP) is a kind of thin hyaline-like cartilage layer covering the cranial and caudal ends of NP, with its thickness estimated to be 0.1–1.6 mm. As we know, blood vessels only enter the outer space of the annulus fibrous, and the CEP thus acts as the main supply of nutrients and oxygen for inner cells of the disc by permeation (Figure 1) (Huang et al., 2014). However, persistent chronic inflammation of CEP elicited by pathological factors can lead to CEP damages, which can further result in chondrocyte aging, loss of cell phenotype, and reduced differentiation capacity by the released inflammatory cytokines due to the degradation of disc ECM (Dudli et al., 2016; Moore, 2006). In 1988, Modic et al. detected and defined the different manifestations of CEP inflammation on MRI as Modic changes, and further divided them into three types: Modic type I (inflammatory phase - T1 low signal, T2 high signal), Modic type II (fat phase - T1 high signal, T2 high signal) and Modic type III (sclerotic phase - T1 low signal, T2 low signal) (MODIC et al., 1988).

As the knowledge of IVDD advances, researchers find that CEP inflammation is closely related to the development of IVDD (Li et al., 2010; Wang et al., 2017; Wong et al., 2019). The disturbance of CEP will cause pathological damage to the development of IVVD, because the main supply of nutrition and oxygen for the IVD was restricted and damaged during CEP inflammation. In addition, IVDD caused by injury, inflammation, and infection will lead to CEP inflammation as well (Crockett et al., 2017; Ganko et al., 2015; Weiler et al., 2005). However, the causal relationship and underlying mechanisms between CEP inflammation and IVDD are rarely reported. To advance our knowledge on the development of IVDD, this study mainly summarizes on how CEP inflammation is interconnected to the development of IVDD.

## 2 Current knowledge on intervertebral disc degeneration

The volume of NP cells (NPCs) occupied in IVD only account for 1% of the IVD, but their roles are indispensable for the physiology and biomechanics of IVD (Silwal et al., 2023). NPCs are important for regulating the metabolism of NP ECM, where collagen type II (Col II) and proteoglycan are the key contents (Zhang G. Z. et al, 2021). The main function of those structures is to cushion and decentralize the pressure suffered by the spine during loading (Chang et al., 2022). Therefore, the physiological function of NP ECM is crucial for maintaining the structure and function of the IVD, as well as the development and progression of IVDD (Xing et al., 2021), because the disturbance of ECM metabolism will lead to the loss of proteoglycan and water content of the IVD (Roh et al., 2021). Many studies have shown that inflammation, oxidative stress, abnormal mechanical load, and other pathological factors are involved in the development of IVDD (Huang et al., 2023; Kang et al., 2023; Wu et al., 2022). For example, inflammatory mediators such as IL-1β can disrupt the metabolic homeostasis of the ECM (Wang et al., 2020). Mitochondrial dysfunction can also contribute to the development of IVDD by producing reactive oxygen species (ROS), where excessive ROS activates the expression of various oxidative stress biomarkers, including phospholipase and NO, which can further result in DNA damage, lipid metabolism, and protein synthesis disorders in IVD (Chen et al., 2024). Of note, abnormal mechanical stress can accelerate ECM degradation and IVDD development as well (Zhou et al., 2024).

**FIGURE 2:**
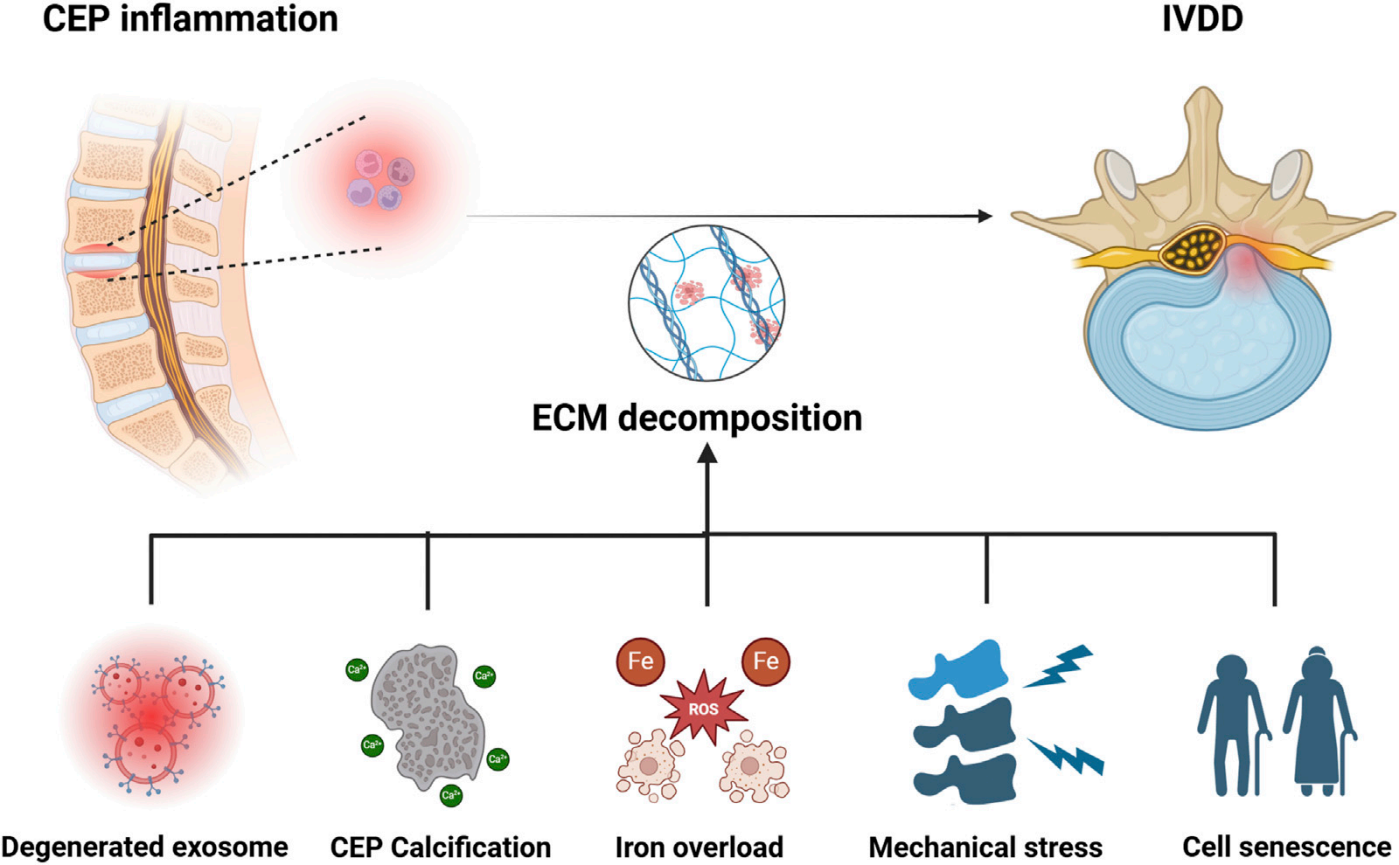
Pathological mechanisms of IVDD caused by CEP inflammation. Degenerated exosome, calcification of CEP, iron overload, mechanical stress, and senescence of NP cells are all involved in the development of CEP inflammation induced IVDD. Image created with BioRender (www.biorender.com).

As we know, CEP acts as a key component of the spine. The normal physiological function of CEP is important in maintaining spinal cord function and providing nutritional support for IVD (Maatta et al., 2016). The inflammation and degeneration of CEP can downregulate its physiological function, and can further lead to the development of IVDD (Ma et al., 2024). However, pathological factors that can result in inflammation and degeneration of the CEP are not clearly presented. Scholars reported that abnormal exosome synthesis, cell calcification, iron overload, abnormal mechanical load, cell senescence, and other factors are accountably involved in this pathological process, which jointly or separately destroy the metabolic balance and accelerate the development of IVDD (Figure 2). Therefore, it is particularly important to explore the pathological factors that lead to the degeneration of NPCs.

## 3 Potential mechanisms underlying IVDD induced by CEP inflammation

### 3.1 Exosomes degeneration

Progenitor cells in human CEP can differentiate into osteoblasts, adipocytes, and chondrocytes (He et al., 2018), which are named as cartilage endplate stem cells (CESCs) by researchers. As reported, CESCs can maintain the integration of structure and function of CEP by paracrine activation of the SDF-1/CXCR4 and ERK1/2 signal pathways, which pathways are conducive for accelerating the proliferation and regeneration of NPCs. Moreover, CESCs can release a type of extracellular vesicle called CESCs-derived exosomes (Phinney and Pittenger, 2017). As demonstrated, those exosomes play important roles in intercellular communication by transferring certain proteins and RNAs, to control inflammation and prevent tissue degeneration by inhibiting cell apoptosis (Fan et al., 2020; Xu et al., 2019). What’s more, such exosomes can not only induce the CESCs to differentiate into NPCs, but also can promote the proliferation of NPCs, which altogether delay the development and progression of IVDD (Luo et al., 2021a). However, CEP inflammation significantly weakens the function of exosomes released from CESCs, and accountably aggravates the progression of IVDD (Luo et al., 2021b).

**FIGURE 3:**
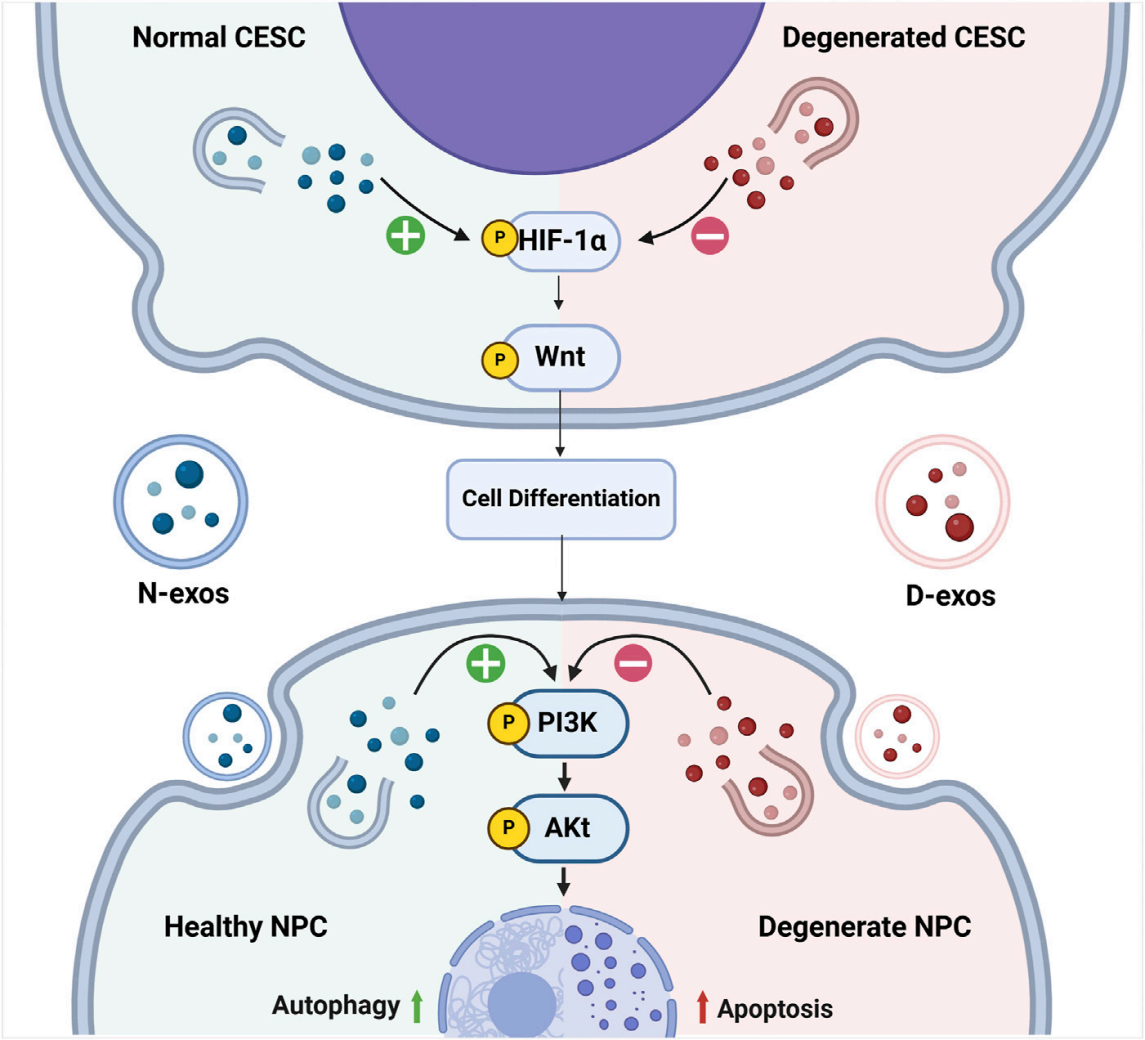
The changes of associated pathways elicited by CEP inflammation during the development of IVDD. Normal CESC-derived exosomes (N-exos) can effectively activate the PI3K/AKT signaling pathway in NPCs and HIF-1α/Wnt signaling pathways in CESCs, which further enhances autophagy, alleviates NPC cell apoptosis, and improves NPC differentiation. While the degenerated CESC-derived exosomes (D-exos) do the opposite, resulting in decreased number of the NPCs. Image created with BioRender (www.biorender.com).

Accordingly, persistent inflammation can result in the CESCs to degenerate, which changes the contents of those secreted exosomes, where such exosomes are named as degenerated CESC-derived exosomes (D-exos) by researchers. Compared to normal CESC-derived exosomes (N-exos), the efficacy of D-exos on reversing IVDD is greatly weakened, and the mechanisms are as follows: firstly, N-exos can activate autophagy associated PI3K/Akt signaling pathways to inhibit the apoptosis of NPCs; secondly, N-exos can significantly reduce apoptotic proteins such as cleaved caspase3 and Bax in NPCs, while anti-apoptotic protein Bcl-2 is increased in such process (Luo et al., 2021b). Meanwhile, N-exos can also activate HIF-1α/Wnt signaling pathways to promote the transformation of CESCs into NPCs by an autocrine mechanism (Luo et al., 2021a). Compared to N-exos, D-exos carried fewer anti-apoptotic proteins, their ability to activate PI3K/AKT signaling pathways in NPCs and HIF-1α/Wnt signaling pathways in CESCs is also decreased. Those changes in D-exos downregulate NPCs autophagy and cause the NPCs to develop apoptosis, resulting in reduction of NPCs (Figure 3) (Luo et al., 2021b).

Studies also find that N-exos can affect the progression of IVDD by their carried miRNAs. For example, miR-532-5p released by N-exos can downregulate the expression of caspase-3, caspase-9, caspase-8, and MMP-1 in NPCs, while upregulate the expression of collagen type I (Col I), collagen type II (Col II) and proteoglycan. Besides, miR-532-5p can inhibit the release of inflammatory factors such as IL-6 and IL-1β by targeting RASSF5 (Zhu et al., 2020). MiR-125-5p secreted by N-exos can inhibit the apoptosis of nucleus pulposus tissue by downregulating the gene expressions of SUV39H1, Bax, MMP13, and p62, while upregulate the gene expressions of Bcl2, ACAN, LC3-II/I. However, the expression levels of miR-532-5p and miR-125-5p are downregulated in D-exos (Chen and Jiang, 2022).

### 3.2 CEP calcification

Calcification of the CEP can also lead to the development of IVDD. As we mentioned previously, the degeneration of CEP can result in the release of proinflammatory cytokines, such as tumor necrosis factor and interleukins (Brisby, 2006). Moreover, those inflammatory factors can increase the risk of the CEP to calcification and chondroid tissue formation as well (Bessueille and Magne, 2015; Joshi et al., 2016). Researchers find that the TNF-α can predispose vascular smooth muscle cells (VSMCs) to form calcium deposits (Guerrero et al., 2012; Lencel et al., 2011; Masuda et al., 2013), where the release of bone morphogenetic protein 2 (BMP-2), a potent osteosynthesis factor, is revealed to accelerate the calcification of involved tissues (Nakagawa et al., 2010). Furthermore, TNF-α can also reduce the levels of extracellular inorganic pyrophosphate (PPi), which is reported to be a potent endogenous inhibitor of calcification (Lencel et al., 2011; Zhao et al., 2012). In addition, some researchers have confirmed that the inflammation of CEP is closely associated with the calcification of IVD (Joshi et al., 2016).

**FIGURE 4:**
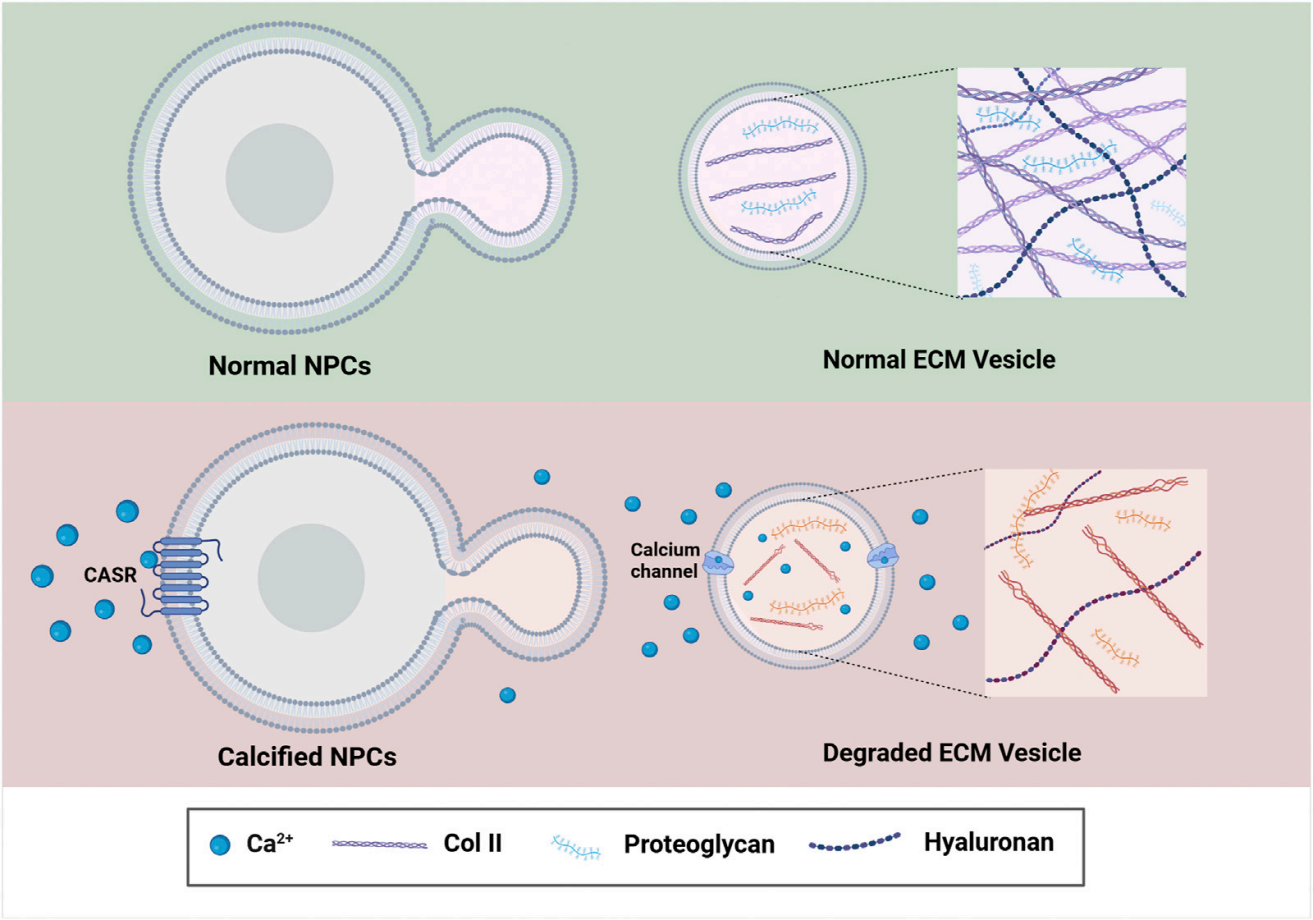
The underlying mechanism of CEP Calcification associated with the development of IVDD. Increased Ca^2+^ in the IVD microenvironment enhance the expression of CASR, which further reduces ECM synthesis by downstream signaling and finally contributes to the calcification of the IVD. Image created with BioRender (www.biorender.com).

Besides, the calcified CEP can affect the nutrition and oxygen exchange of the IVD as well. As we described previously, the intervertebral disc is an avascular tissue structure, where capillaries originated from the vertebral body terminate at the periphery of intervertebral disc. Therefore, nutrients of the NP cells needed, such as glucose and oxygen, can only be transported by diffusion through the CEP (Sakai and Grad, 2015). Due to this anatomy of the disc, the permeability of the CEP can be significantly decreased when the CEP becomes calcified, those changes will further decrease the glucose and oxygen delivered to the IVD, and further result in intervertebral disc degeneration over time. Extracellular Ca^2+^ content is found to elevate after CEP calcification in the microenvironment of IVD as well. As reported, the elevated Ca^2+^ can activate the extracellular calcium-sensing receptor (CASR), a C-g protein-coupled receptor (GPCR), to regulate the synthesis and secretion of parathyroid hormone (Brown, 2013). While the activation of CASR is responsible for the aggravation of denatured NP cells (Grant et al., 2016), as decreased secretion and accumulation of beneficial matrix molecules, such as Col II and proteoglycans, can be observed in the NP cells during this process (Lakstins et al., 2021; Wong et al., 2019; Zhao et al., 2007). Besides, ECM synthesis-catabolism imbalance will be developed within the NP cells during CEP calcification, where more ECM is degraded than produced (DeLucca et al., 2016; Kim et al., 2009). Over time, the water content within the IVD is significantly decreased, and intervertebral disc subsidence will then occur (Figure 4) (van Uden et al., 2017).

Moreover, calcified IVDs are more likely to rupture and herniate than non-calcified IVDs and normal IVDs, because the loading stress of the spine is abnormally distributed when CEP gets calcification, which greatly increases the risk of developing vertebral fractures and fissures (Fearing et al., 2018). In addition, calcified intervertebral disc can result in caseous degeneration due to calcified deposits, which can even be spontaneously liquefied in the late stage. Calcification can also cause damage to the annulus fibrosus or surrounding soft tissue, and inflammation can further accelerate this process, which altogether lead to the development of IVD herniation (Court et al., 2018; Yu et al., 2020; Yue et al., 2016). Besides, Calcified tissue in CEP can destroy the microstructure of bone trabeculae, which is another factor that leads to endplate fracture (Crockett et al., 2017). Moreover, the calcified tissue in CEP can result in anisotropic cracks of CEP as well. Those cracks in CEP can further accelerate the loss of water content in the IVD, while simultaneously increase the flow of inflammatory cytokines and cells (Crockett et al., 2017).

### 3.3 Iron overload

Iron is considered as the most abundant trace element in the human body. Nowadays, various studies have proved that iron’s abnormal deposition is correlated to the development of IVDD and CEP inflammation (Jing et al., 2021a; Jing et al., 2021b; Nieuwenhuizen et al., 2013). Unfortunately, our human body lacks the ability to get rid of excess iron, which causes the irons to be gradually accumulated as we become aged (Tian et al., 2022). Recent studies report that chondrocyte senescence and degeneration are largely involved in iron toxicity caused by oxidative stress and iron overload (Li et al., 2023; Yao et al., 2021). Accordingly, the balance of iron metabolism within cells is regulated by iron transporters DMT1 and FPN1, where DMT1 serves as the transporter for taking up iron and FPN1 is responsible for facilitating the outflow of iron (Galy et al., 2023), while ferritin is responsible for storing iron (Zeidan et al., 2021). Some studies have shown that when CEP is inflamed, the inflammatory cytokines IL-6 and TNF-α can upregulate the expression of DMT1 while decreasing the expression of FPN1 in cells (Li et al., 2021; Urrutia et al., 2013), which changes significantly elevate iron depositions in the body. Meanwhile, IL-6 and TNF-α also increase the expression of hepcidin (Frazier et al., 2011), an another factor that destroys iron homeostasis (Stephenson et al., 2014).

Iron overload can produce large amounts of reactive oxygen species (ROS) by electron transfer in the mitochondrial oxidative respiratory chain (Mishima et al., 2022), where excess ROS is closely associated with mitochondrial dysfunction and DNA damage (Chen et al., 2020). Scholars have proved that iron overload can aggravate oxidative stress and mitochondrial function in a dose-dependent manner (He Q. et al, 2023), which further induces iron-dependent cell death (Ferroptosis) of the CEP and NP cells (Dixon et al., 2012). Ferroptosis is a recently discovered mode of cell death, a process characterized by lipid peroxidation catalyzed by iron ions (Tang et al., 2020). Nowadays, many reports have pointed out that ferroptosis is closely related to IVDD (Zhang P. et al, 2023; Zhu et al., 2023). In a TBHP-induced oxidative stress model, the increased changes of ferroptosis-associated marker and lipid peroxidation are positively correlated with IVD cell degeneration (Fan et al., 2023).

Moreover, excess iron in NP cells can activate transcription factor 3 (ATF3), a positive regulator of ferroptosis (Fu et al., 2021; Wang et al., 2019), while inhibit glutathione peroxidase 4 (GPX4) and cystine/glutamate antiporter SLC7A11 (xCT) (Wang W. et al, 2022; Zhang et al., 2020), which changes significantly induce ferroptotic cell death in the NP cells. Another major cause of ferroptosis is lipid peroxidation (Yang et al., 2020). In the human body, fatty acids are the major components used for the synthesis of phospholipid bilayers of cell membranes, and those components are also major substrates for energy metabolism (Bersuker et al., 2019). When Fe^2+^ are largely accumulated in the cytoplasm, toxic lipid ROS are produced (Doll et al., 2016), where the polyunsaturated fatty acids (PUFA) of phospholipids in the cell membrane are more likely to be bound by ROS due to its highly expressed active hydroxyl radicals (Kayagaki et al., 2021; Yan et al., 2021). Lipid peroxidation driven by free radicals produces lipid oxygen peroxides (LOOH), a enzyme that can damage the continuity of the lipid bilayer by disrupting the integration of cell membrane, thereby inducing ferroptosis of the NP cells (Fan et al., 2023).

Iron overload is also correlated with aging and degeneration of the chondrocytes (Hou et al., 2016; Jing et al., 2021a). Although abnormal iron ions have played some beneficial roles in increasing the expression of Col10 and Runx2 to promote the formation and mineralization of CEP (Wang W. et al, 2022; Yao et al., 2019). However, excessive iron deposition upregulates matrix metalloproteinases, such as Mmp3 and Mmp13 in CEP cells, which evidently reduces the expression of Sox9 and Col II (Wang W. et al, 2022), thus facilitating the breakdown of cartilage matrix and expediting the deterioration of CEP, and finally contributes to the development of IVDD (Yuan et al., 2019).

### 3.4 Mechanical changes

Non-physiological mechanical load is one of the important factors known to affect IVDD as well (Belavy et al., 2016; Vergari et al., 2016; Wuertz et al., 2009). While the relationship between mechanics and biology is complex, it has been established that CEP inflammation can lead to microfracture of the cartilage endplate and abnormal mechanical loading (Feng C. et al, 2018; Xiao et al., 2018; Zheng et al., 2019). As reported, The presence of inflammatory mediators such as TNF-α increase the sensitivity of the CEP to mechanical loading, thereby aggravate mechanical stresses applied to the CEP, which contribute to the development of microfractures (Din et al., 2021). Besides, the persistent production of proinflammatory substances can expand the broken endplate, or even extend to the whole NP and AF (Crockett et al., 2017; Kameda et al., 2017). In addition, inflammation increases vascular permeability of the damaged CEP, which can allow low-virulence bacteria to infiltrate into the IVD along the newly formed capillaries, and this is another factor that is associated with the development of IVDD (Hernandez et al., 2020). Moreover, the existence of inflammatory mediators like TNF-α can aggravate the outcomes of mechanical stress exerted on the IVD (Liu et al., 2023; Snuggs et al., 2021; Zhang A. et al, 2023). Under the stimulation of inflammatory factors, abnormally changed osmotic stress elicited by mechanical loading can more fiercely disrupt the F-actin structure and cell volume of the NP cells (Kletsas et al., 2014), even a complete absence of intracellular F-actin in the NP cells can be observed (Kletsas et al., 2014). By *in vitro* experiments, researchers reveal that those morphological and biophysical characteristics of the NP cells suffered are irreversible, even those cells are cultured in normal conditions after the discontinuation of inflammatory stimulus (Jacobsen et al., 2021; Kletsas et al., 2014).

Interestingly, recent relevant studies reveal that cells isolated from IVD tissue with inflammation show different responses to mechanical stress compared to cells isolated from normal IVD tissue (Mainardi et al., 2022; Tavakoli et al., 2020). It has been reported that normal AF cells can maintain proteoglycan production when mechanical stretch strains of low intensity (1%) and physiological frequency (1 Hz) are applied (Pratsinis et al., 2016). Besides, NP cells can observe an anabolism response under low-to-moderate intensity stretch, and only higher intensities promote a catabolism response (Fearing et al., 2018). However, Inflammatory factors such as TNF-α would alter the sensitivity of IVD to normal mechanical stress, thereby nullifying this beneficial effect under low-to-moderate mechanical stretch (Torre et al., 2018). Increased evidences have shown that stretch and inflammatory signaling are interacted in the degeneration process of AF cells (Linder et al., 2021; Ramanathan et al., 2022; Wang A. G. et al, 2022), because scholars discover that inflammatory signalings can alter the cytoskeletal mechanical transduction of the IVDs in recent studies. In AF cells, the expression of F-actin stress fiber of α-tubulin is found to be enhanced following TNF-α treatment, which may predispose the AF cells susceptible to stress of mechanical strain (Gonçalves et al., 2022). Moreover, mechanical stretches suffered by AF cells can activate inflammatory receptors such as TLR as well (Mohd Isa et al., 2022), while the changes of those receptors are demonstrated to be correlated with cytoskeletal remodeling and biomechanical alterations of the NP cells.

### 3.5 Cell senescence

Senescence is also another factor that leads to the development of IVDD. Currently, multiple studies have showed close relationships between senescent NP Cells and IVDD (Zhang K. et al, 2021; Zhang et al., 2024). Short-term exposure to senescence is harmless due to the clearance of the immunological effect, but long-term exposure will lead to uncontrolled chronic inflammation owing to the disrupted homeostasis of the immune system in this condition (Calcinotto et al., 2019; Herranz and Gil, 2018). As researchers reported, chronic inflammation of CEP is recognized as a characteristic feature of senescence (Baechle et al., 2023; Salvioli et al., 2023). In such pathological status, overproduced inflammatory factors such as IL-1β and TNF-α are observed and can subsequently lead to senescence of the NP cells (Capoor et al., 2021; Zhu et al., 2022). Studies have shown that endplate chondrocytes are involved in the regulation of cell proliferation, differentiation, and senescence through the Hippo-Yes-associated (YAP*)* pathways (Pan et al., 2021; Sladitschek-Martens et al., 2022), and the decrease of YAP1 can further stimulates the senescence of IVD cells during inflammation in the CEP (Kong et al., 2023).

Besides, increased inflammation of the cartilage endplate can result in elevated expression of SA-β-Gal, an age-related protein (Purmessur et al., 2013). TNF-α generated during inflammation also induces downregulated expressions of proteoglycan, Col I, and Col II in the NP cells as well. For instance, Kang et al. found the proportion of aging markers (e.g., P16 and p53) in TNF-α-induced NP cells were increased in an IVDD inflammation model (Li et al., 2019). In addition, senescence can also impact the cell cycle of the NP cells, where senescence induced by TNF-α is more likely to make the NP cells to stay in G0/G1 phase. While S phase is decreased during this process, indicating an occurrence of cell growth arrest. Besides, the proliferation of NP cells is also inhibited after senescence induced by TNF-α (Li et al., 2019). In addition, other pro-inflammatory mediators, such as IL-2, IL-4, IL-8, IFN-γ, and prostaglandin 2, are also responsible for aggravating the aging of the NP cells, which combinatorially lead to the development of IVDD (Huang et al., 2018; Miyagi et al., 2022; Risbud and Shapiro, 2013).

**FIGURE 5:**
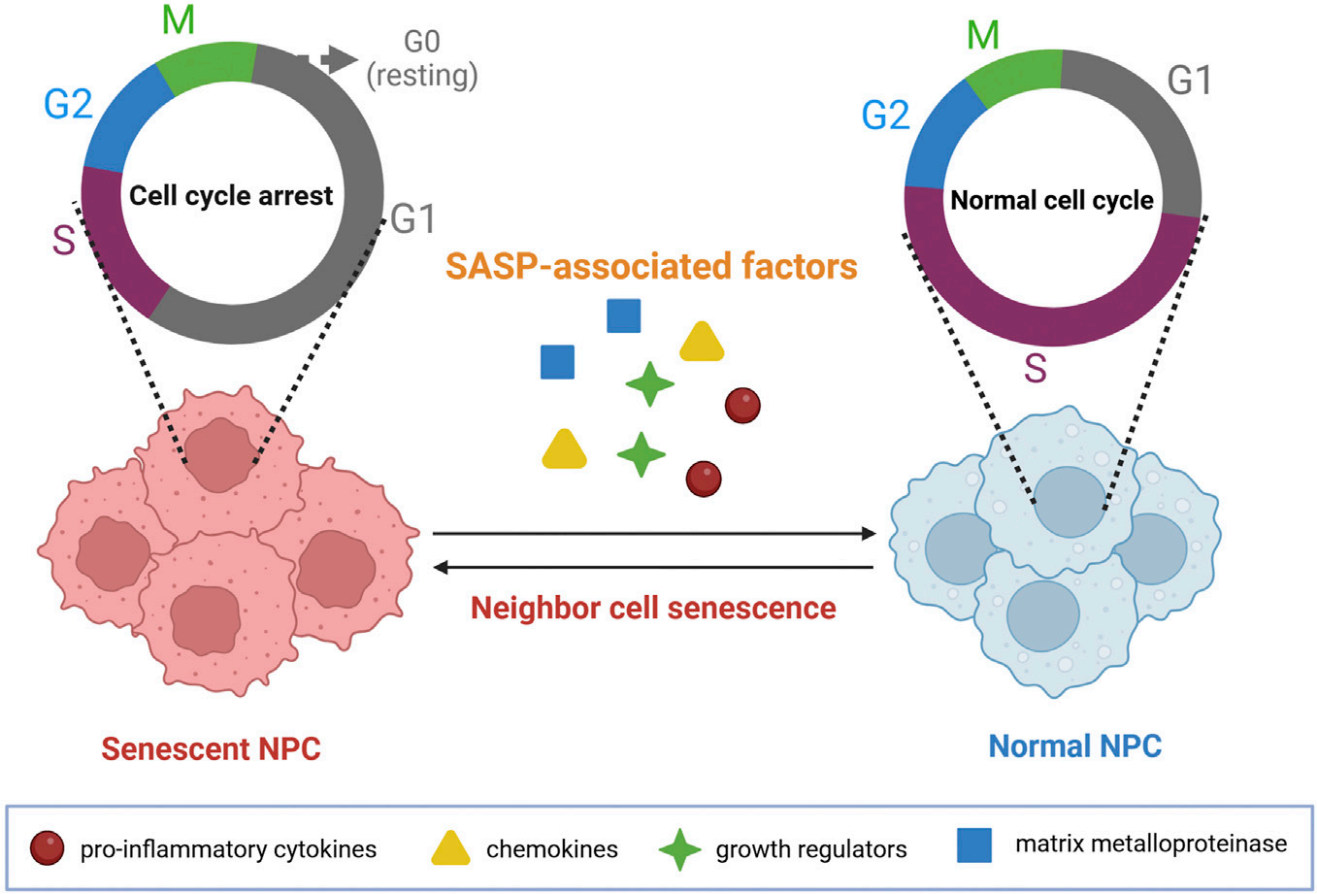
SASP induces cell cycle arrest within NP cells. SASP-associated factors, such as proinflammatory cytokines, chemokines, growth regulators, and matrix metalloproteinase, can significantly lead to the senescence of neighboring cells by the paracrine pathways. Image created with BioRender (www.biorender.com).

As cells becoming aged, a variety of pro-inflammatory cytokines, chemokines, growth factors, and MMPs can be secreted, which are then named as senescence-associated secretory phenotype (SASP) by scholars (Patil et al., 2019). Accordingly, increased production of MMPs can lead to hydrolysis of Col II and proteoglycan around the NP cells (Bedore et al., 2014; Qin et al., 2022), which disrupts the metabolic balance of the NP ECM, predisposing the human body easier to develop IVDD (Chen et al., 2022; Cherif et al., 2020). SASP can induce senescence of the neighboring cells by autocrine and paracrine mechanisms as well (He X. et al, 2023; Herranz and Gil, 2018). Moreover, the cell division cycle can be prolonged by SASP through an autocrine pathway, which makes the NP cells to more likely be stayed in a static phase (G0 phase) (Ji et al., 2023), resulting in decreased S and M phases (Figure 5) (Georgilis et al., 2018; Hubackova et al., 2015). Noteworthily, the cessation of cell cycle induced by SASP is often irreversible (Acosta et al., 2013). Besides, other cytokines such as IL-6, monocyte chemotactic protein-1, and IGF binding proteins can also make some contributions to the development of NP senescence by paracrine mechanisms (Freund et al., 2010; Jeon et al., 2017).

### 3.6 Others

As reported, miRNAs are also discovered to involve in the development of IVDD induced by CEP inflammation (Chandan et al., 2020). The expressions of miRNAs such as miR-640 and miR-194 are upregulated during IVDD, which changes evidently reduce the chondrogenic differentiation of CESCs and increase the osteogenic differentiation of CESCs (Cazzanelli and Wuertz-Kozak, 2020; Ma et al., 2024). For instance, Dong et al. found that upregulated expression of miR-640 is observed in the inflamed IVD cells, and stimulation of IVD cells with TNF-α and IL-1β can reciprocally upregulate miR-640 expression as well. In addition, miR-640 is found to be involved in NP cell apoptosis, where upregulation of MMP-3 and MMP-9 and downregulation of proteoglycan and Col II of the NP cells can be observed (Dong et al., 2019). Moreover, upregulation of miR-194 of the NP cells induced by IL-6 and TNF-α is revealed to upregulate the expression of CUL4A and CUL4B genes, which are then demonstrated to be positively correlated with the severity of IVDD (Chen et al., 2019). Except for the aforementioned miRNAs, miR-194 and miR-515 are reported to involve in the development of IVDD by degrading chondroitin sulfate synthase CHSY-1/2/3 of the NP cells (Hu et al., 2017).

**FIGURE 6:**
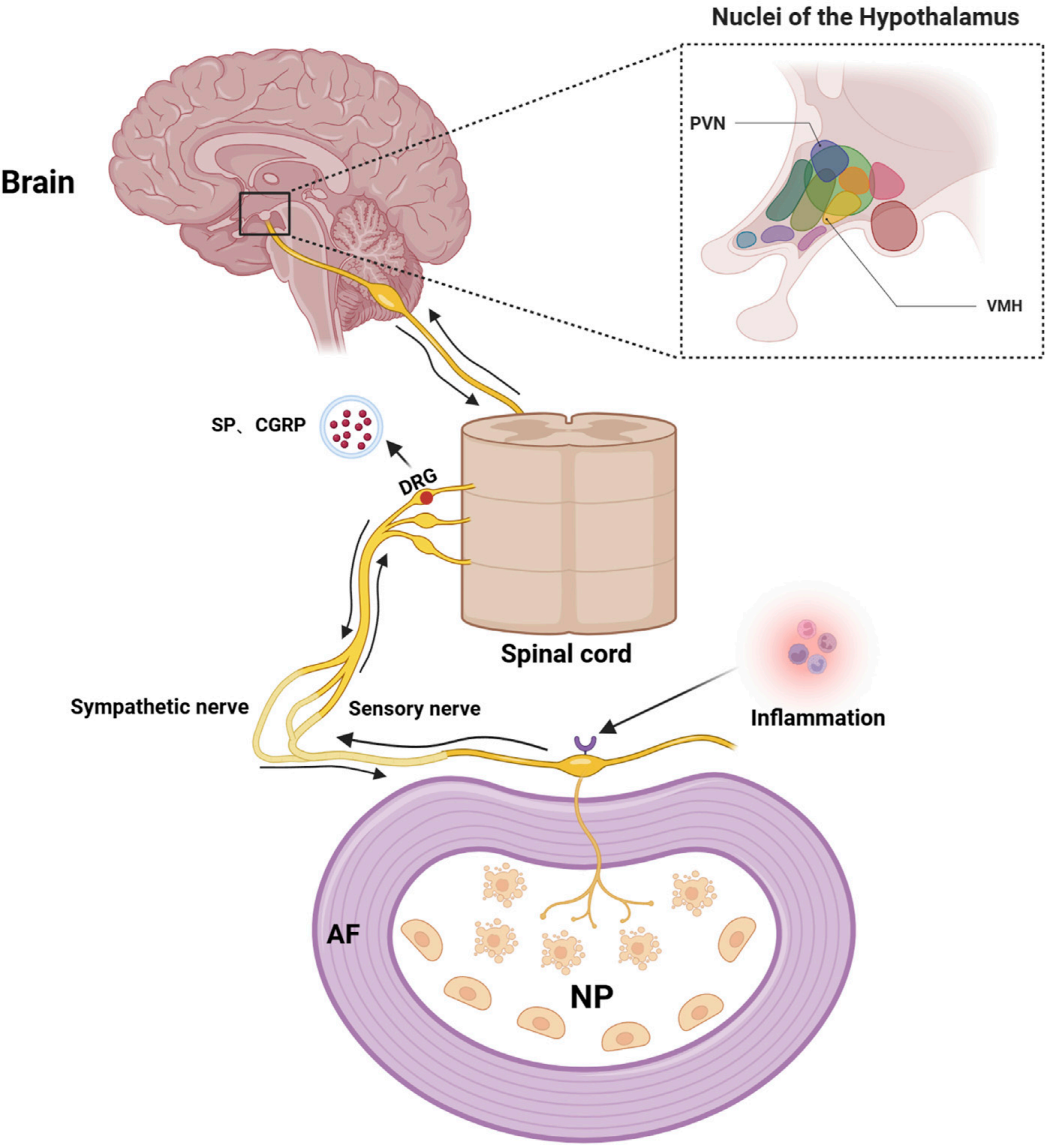
Schematic illustration of the IVD-DRG/sympathetic nerve-VMH/PVN axis. TNF-α and IL-1β evoke IVDD pain by reacting with their nociceptors of the local nerve endings when CEP is inflamed, which transmits pain sensations to the DRG and further increases secretion and transport of neurotransmitters into the corresponding levels of IVD via their receptors. Those neurotransmitters can then affect the neurons in the central nervous system, such as the ventromedial hypothalamic nucleus (VMH) and paraventricular nucleus (PVN) of the hypothalamus, to reciprocally regulate the IVDD pain. Image created with BioRender (www.biorender.com).

In recent years, abnormal changes in pain-related neural pathways have aroused great interest in scholars, and those abnormal changes are discovered to be intimately correlated with the development of IVDD (Kim et al., 2020). As we know, substance P (SP) and calcitonin gene-related peptide (CGRP) are important neurotransmitters to regulate pain perception and transmission in the nervous system. Interestingly, positive sensory and sympathetic markers of the nervous systems are detected in degenerated IVD tissues (Sainoh et al., 2014; Song et al., 2020), where increased CGRP expression is detected in painful and degenerative discs (Ahmed et al., 2019). Blocking SP or CGRP production in dorsal root ganglion (DRG) neurons of the IVD can relieve pain symptoms significantly (He et al., 2020). Of note, TNF-α and IL-1β are considered to be the main inflammatory mediators to produce new nerve fibers that lead to IVD pain (Risbud and Shapiro, 2013). When CEP is inflamed, TNF-α and IL-1β infiltrate the annulus fibrosus through the damaged cartilage endplate, which promotes local nerve endings to infiltrate into the nucleus pulposus and induce pain by stimulating nociceptor responses (Sun et al., 2022). Then, those evoked pain sensations transmit to the DRG via generated electronic potential by nociceptors, which will then result in increased secretion and transport of neurotransmitters, such as SP and CGRP, into the corresponding levels of IVD via their corresponding receptors (Seidel et al., 2013; Wise et al., 2020). Those neurotransmitters can affect the neurons in the central nervous system as well, including the ventromedial hypothalamic nucleus (VMH) and paraventricular nucleus (PVN) of the hypothalamus, where excited VMH and PVN can reciprocally increase the activities of sympathetic nervous system and DRG, by secreting corresponding neurotransmitters (Sun et al., 2022) (Figure 6).

## 4 Perspectives and future directions

IVDD is a kind of degenerative disease of the spine, and severe social and economic burdens can be caused by this disease if left untreated. In healthy IVD, the balance between anabolism and catabolism of the ECM is important for the stability of the spine. However, pro-inflammatory factors produced by CEP inflammation significantly disrupt this balance, and ultimately result in the development of IVDD due to the loss of ECM (Crump et al., 2023; Zou et al., 2023). To what we have described previously, changes of secreted exosomes, cell calcification, iron overload, mechanical stress, and cellular senescence, are all contributing factors for the development of CEP inflammation-induced IVDD. However, strategies dedicated to treating this disease are remained to advance. Nowadays, exosomes are becoming more and more welcomed for treating degenerated diseases (Wei et al., 2019; Zhu et al., 2018), and exosomes can also be engineered to render them with special functions, which include virus transfection, ultrasound-assisted drug loading, and cointubation with certain proteins (Luo et al., 2021b). Therefore, engineered exosomes can be a promising direction for treating IVDD.

Except for exosomes, MSC transplantation has also detected beneficial roles in treating IVDD. By injecting nanofiber sponge-like microbeads with loaded MSCs, the cell phenotype of nucleus pulposus can be maintained by anti-microRNA-199a released by MSCs, where calcification can also be inhibited simultaneously (Feng et al., 2020; Hu et al., 2024). Furthermore, iron chelators (DFO), antioxidants (NAC), and ferroptosis inhibitors (Fe-1) are also good alternatives for preventing the degeneration of endplate chondrocytes that are caused by iron overload (Wang W. et al, 2022). As demonstrated, DFO prevents the downregulation of GPX4 and SLC7A11 by reducing iron load, while NAC and Fe-1 inhibit oxidative stress and ferroptosis (Jeney, 2017; Martacic et al., 2018). Infliximab is discovered to be powerful in inhibiting the production of pro-inflammatory cytokines by binding and isolating TNF-α, and thus can be another alternative for treating IVDD (Syversen et al., 2021). In addition, some Chinese medicine extracts, such as Resveratrol, can be helpful in reversing the harmful effects elicited by inflammatory cytokines (e.g., IL-1β and TNF-α) on the NP cells as well, where ROS elimination and G0/1 cell cycle promotion effects are observed (Li et al., 2019).

In addition, miRNA-based therapies for IVDD have also been developed. For example, an injectable MMP-degradable hydrogels containing miR-29a was developed for inhibiting IVD fibrosis and reversing IVDD (Feng G. et al, 2018). Interestingly, chondroitin sulfate transplantation can ameliorate the decreased chondroitin sulfate synthesis caused by miR-194 and miR-515, which further reverses the development of IVDD (Luo et al., 2023). Besides, Enhancing the function of neurotransmitters in focal environment of the IVD, such as exogenous administration of NPY or VIP, or inhibiting harmful neurotransmitters by local antagonisms of CGRP or SP, are potential approaches for future treatment of IVDD (Sun et al., 2024). Nevertheless, more treatment strategies specifically targeted to the IVDD are still needed to explore in the future.

## 5 Conclusion

CEP plays an important and indispensable role in maintaining the function of the intervertebral disc, as this structure not only cushions and distributes the mechanical load of the spine, but also is the key supply for providing nutrition and oxygen for the discs. However, CEP becomes inflamed when suffered from uncontrolled factors, where inflammatory factors such as IL-1β and TNF-α disrupt the metabolic balance of ECM synthesis and further lead to the degradation of intervertebral discs. Moreover, CEP inflammation can influence exosome function of CESCs, promote IVD calcification, induce iron overload of NP cells, increase IVD sensitivity to mechanical stress, and induce cellular senescence as well, which factors all accountably contribute to the development of IVDD. Meanwhile, this paper has also reviewed relevant miRNAs and pain-related neural pathways involved in this process, such as miR-640 and CGRP. At last, potential treatment strategies are also reviewed in the perspective section. We hope this review can not only provide new ideas and references for treating IVDD, but also inspire researchers in this field to develop more advanced strategies in the future.
